# CT imaging of a rare case of persistent fifth aortic arch in newborn

**DOI:** 10.1259/bjrcr.20150048

**Published:** 2016-12-15

**Authors:** Nicolò Schicchi, Giacomo Agliata, Andrea Giovagnoni

**Affiliations:** ^1^ Radiologia Pediatrica e Specialistica, Ospedali Riuniti di Ancona, Ancona, Italy; ^2^ Scuola di Specializzazione in Radiodiagnostica, Università Politecnica delle Marche, Ancona, Italy

## Abstract

Congenital persistence of the fifth aortic arch is an unusual, often misdiagnosed and underestimated pathological finding. It is usually associated with other cardiac or vascular anomalies, which makes every case quite unique in its clinical presentation and treatment. Our subject was a newborn (1-month-old male) who was referred to our hospital from a peripheral centre owing to difficulty in obtaining a clear diagnosis with traditional means (echocardiography). He presented with Type II left-sided malformation (atresia or interruption of the superior arch with patent inferior arch) and also showed an associated atrial septal defect with left-to-right-shunt. The investigation was carried out with a contrast-enhanced CT scan owing to the serious clinical condition (haemodynamic instability) of the subject that made an MRI examination too hazardous. The study succeeded in plainly depicting the malformation, providing a clear diagnosis and also giving the surgeons (especially with the assistance of three-dimensional volume rendering reconstruction) an accurate anatomical model, which played a crucial role in planning the operation. The ability of a multislice CT scan to rapidly perform a full, panoramic and minimally invasive study of the cardiovascular system is clearly demonstrated in this study. The only downside of this procedure is the use of ionizing radiation on a newborn, although it is justified in this case by the emergent need for a quick diagnosis. Furthermore, a CT scan is characterized by a higher spatial resolution compared with an MRI and for vascular anomalies, a CT scan is often preferred. An MRI is mainly used in case of functional imaging. Exactly for this reason, we planned the procedure in order to maintain the equivalent radiation dose as low as possible [equivalent dose (H) ≤ 1 mSV].

## Summary

Congenital persistence of the fifth aortic arch is an unusual, often misdiagnosed and underestimated pathological finding. It is usually associated with other cardiac or vascular anomalies, which makes every case quite unique in its clinical presentation and treatment. Our subject was a newborn (1-month-old male) who was referred to our hospital from a peripheral centre owing to difficulty in obtaining a clear diagnosis with traditional means (echocardiography). He presented with Type II left-sided malformation (atresia or interruption of the superior arch with patent inferior arch) and also showed an associated atrial septal defect with left-to-right-shunt. The investigation was carried out with a contrast-enhanced CT scan owing to the serious clinical condition (haemodynamic instability) of the subject that made an MRI examination too hazardous. The study succeeded in plainly depicting the malformation, providing a clear diagnosis and also giving the surgeons [especially with the assistance of three-dimensional volume rendering (VR) reconstruction] an accurate anatomical model, which played a crucial role in planning the operation. The ability of a multislice CT (MSCT) scan to rapidly perform a full, panoramic and minimally invasive study of the cardiovascular system is clearly demonstrated in this study. The only downside of this procedure is the use of ionizing radiation on a newborn, although it is justified in this case by the emergent need for a quick diagnosis. Furthermore, a CT scan is characterized by a higher spatial resolution compared with an MRI and for vascular anomalies, a CT scan is often preferred. An MRI is mainly used in case of functional imaging. Exactly for this reason, we planned the procedure in order to maintain the equivalent radiation dose as low as possible [equivalent dose (H) ≤ 1 mSV].

## Clinical presentation

The patient (born in a peripheral hospital) was arrhythmic (bradycardic, corrected QT interval =  0.48 s) since his birth (Apgar score: 8, owing to bradycardia and light respiratory difficulties). No alterations were noted during pregnancy, neither clinical nor by antenatal sonographies. There was no family history of congenital heart disease. Patient was born by a normal delivery. After the detection of a heart murmur, he underwent an echocardiogram that gave rise to the suspicion of aortic coartaction. Afterwards, he was referred to our hospital for further investigations and treatment. During the hospitalization, his clinical condition worsened with the onset of haemodynamic instability, tachyarrhythmia and detection of an arm–leg pressure gradient of 20 mmHg.

## Differential diagnosis

Possible differential diagnoses are other congenital heart and aortic diseases. Because of the presence of a heart murmur (more easily detectable posteriorly over the neonate’s thoracic spine) and an arm–leg pressure gradient, the first hypothesis considered was aortic coarctation. Other possibilities taken into account were aortic stenosis (bicuspid valve) or the more rare aortic malformation, that is, vascular ring.

## Investigation/main findings

Owing to the difficulties in confirming the hypothesis of aortic coarctation with echocardiography and the serious clinical condition of the patient, in agreement with cardiac surgeons, we decided to perform a CT scan. Our choice was justified by the emergency situation, considering the severe haemodynamic instability of the subject there was a need for a prompt diagnosis, although this meant using X-ray on a paediatric patient.

The study was performed with a 64-slice CT scanner (LightSpeed VCT, GE Medical Systems, Milwaukee, WI) before and after the intravenous injection of 6 ml of iodinated contrast agent (ICA) (Iomeron 400, Bracco Imaging SpA, Milan, Italy). Thanks to the anaesthesiological support, the patient was sedated and intubated before the examination to limit motion and respiratory artefacts. We did not use the prospective electrocardiogram gating because of the tachyarrhythmia and the need to reduce the radiation dose as much as possible. With this aim, we used a low-dose protocol consisting of only two acquisitions, one before and one after the injection of ICA. In particular, the second one was characterized by specific setup parameters of CT scan: 80 kV, 250 mAs, 0.6 mm slices. Our experience shows that, by following our method, it is possible to perform a diagnostic examination with exposure to very low radiation dose (H ≤ 1 mSV). After the acquisition of axial images, we obtained through post-processing multiplanar, maximum intensity projection and VR reconstructions. The procedure took approximately 15 min.

The main finding of our study was the presence of a persistent fifth aortic arch (PFAA), a rare pathological condition that is usually divided into three types.^1–4^ In Types I and II, the PFAA is an anomalous arterial branch that connects two different tracts of a normal aorta, usually the distal ascending aorta near the ostium of innominate artery (that derives, with the normal aortic arch, from the fourth embryonic arch) and the descending aorta.^5^ In this case, the PFAA forms a systemic-to-systemic connection by a double-lumen aortic arch. The difference between the two types is that, in Type I, the lumina of both arches are patent, whereas in Type II, there is atresia or interruption of the superior arch. In Type III, the PFAA makes a systemic-to-pulmonary connection, joining the ascending aorta and the left pulmonary artery (that is a derivative of the sixth aortic arch).

**Figure 1.**
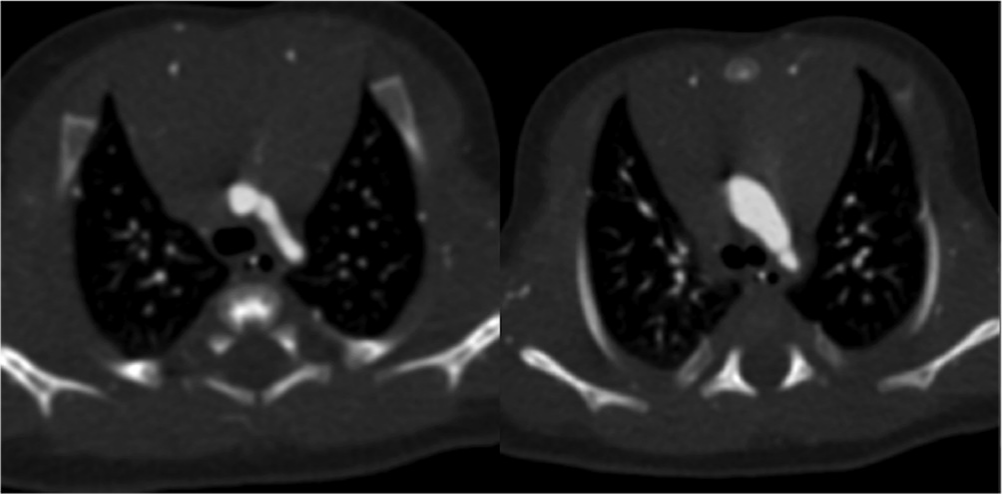
Axial view of two aortic arches. On the left is the superior arch, which is the real one (with a reduced calibre); on the right is the inferior arch, which is the persistent fifth aortic arch (with a greater calibre but hypoplastic in its distal portion).

**Figure 2.**
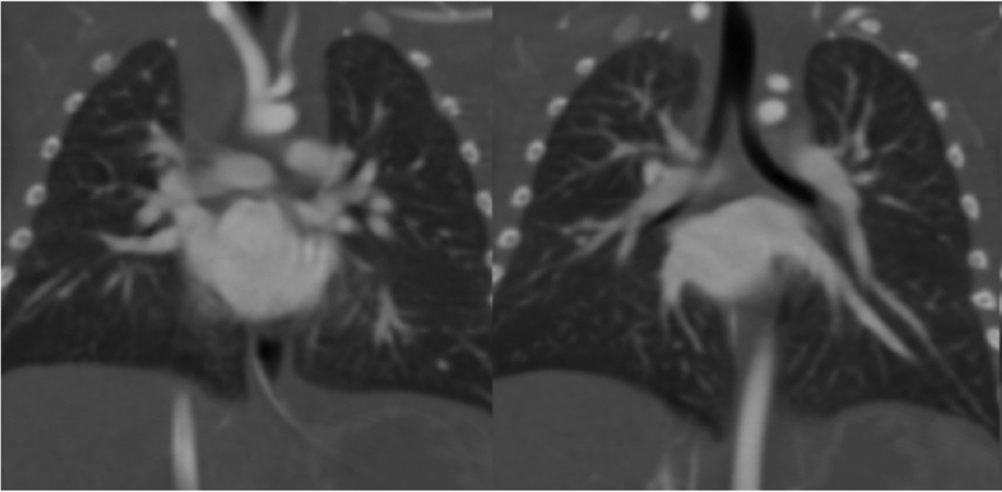
Coronal view of two aortic arches. In these two subsequent images, in addition of what is described in Figure 1, we can see an anonymous arterial trunk arising from the proximal fusion of the two arches and the left carotid artery (of regular calibre) arising from the superior arch.

**Figure 3.**
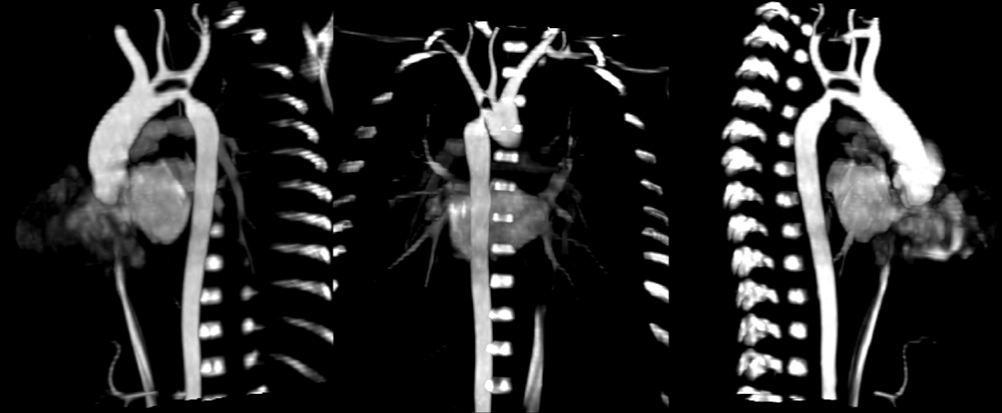
Maximum intensity projection reconstruction. A double aortic arch is shown from a different viewpoint; it demonstrates the rise of supra-aortic branches (the left carotid and subclavian arteries from the superior arch and an anonymous arterial trunk from the proximal fusion of the two arches) and the presence of a hypoplasia in the distal portion of the inferior arch, which continued itself with the aortic isthmus that showed a significant coarctation and a related post-isthmic dilation. Also shown is a distal connection of the upper arch and the aortic isthmus, although it appears essentially atresic.

**Figure 4.**
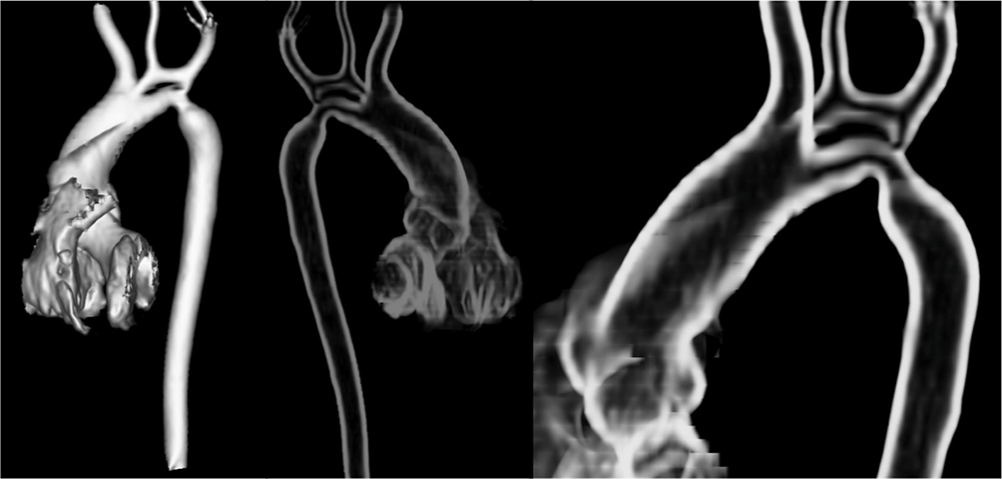
Volume rendering reconstruction. Three-dimensional view of what was seen on maximum intensity projection images.

Our case showed a left-sided double-lumen aortic arch (Type II; Figures 1–4). The superior one was the real aortic arch (with a reduced calibre) from which the left carotid and the left subclavian arteries (both of regular calibre) arose. The anonymous arterial trunk arose from the proximal fusion of the two arches. The inferior one was the PFAA (with a greater calibre but hypoplastic in its distal portion), which continued with the aortic isthmus that showed a significant coarctation and a related post-isthmic dilation. Apparently, a distal connection of upper arch and the aortic isthmus was detected as well, although it appeared very thin/atresic (in fact, a colour Doppler echocardiographic later showed no flux).

PFAA is often associated with other cardiac or vascular anomalies, represented here by aortic coartaction and ostium secundum type atrial septal defect with left-to-right-shunt. In our case, as already reported in the literature, the PFAA was haemodynamically benign compared with other defects; in particular, the coartaction, which was the real cause of the symptoms.^6,7^ The presence of the PFAA implicates, obviously, an important impact on the surgical treatment.

## Treatment

3 days after the CT scan, the child underwent a surgical reconstruction of the aortic arch through end-to-side anastomosis of the two arches, decoarctation by end-to-end technique and closure of the atrial defect with direct suture in extracorporeal circulation and hypothermic (22°C) cardioplegic arrest.

## Outcome and follow-up

The subject was discharged from the hospital 10 days after the successful surgical treatment, with complete resolution of the haemodynamic instability and achievement of patient’s general wellness. The patient had a good condition at further follow-up (follow-up period: 2 years after the operation; echocardiographic examinations: every 3 months).

## Learning points

Contrast-enhanced MSCT is a powerful tool for obtaining a quick diagnosis, especially in emergency situations. If performed properly, it provides a panoramic visualization of the cardiocirculatory system, making it possible to recognize and exhaustively study even complex cardiac malformations, as proven by our experience.

## Consent

Written informed consent was obtained from the patient’s guardians for publication of this case report, including accompanying images.
